# Supplementary material for: Complexome profiling of the Chlamydomonas psb28 mutant reveals TEF5 as an early PSII assembly factor
Source: Plant Cell. 2025 Mar 17;37(6):koaf055. doi: 10.1093/plcell/koaf055 (PMC12164590; doi:10.1093/plcell/koaf055)
Supplement: koaf055_Supplementary_Data [file koaf055_supplementary_data.zip › psb28_interactive.zip!/plots/intensity/qualityPlots/peptides/TEF10a_Cre03.g146167.t1.1.html]

### Cre03.g146167.t1.1

Trivial name: TEF10a  
SeqCoverage [median]: 43.00  
Mass: 24.85 kDa  
  
 

| Sequence | MissedCleavage | Mass [Da] | wtIntensity | muIntensity | Score |
| --- | --- | --- | --- | --- | --- |
| FFSSENLAGMFK | 0 | 1376.64 | 28242.43 | 120711.20 | 251.56 |
| FFSSENLAGMFKAPQ | 1 | 1672.79 | 7226.18 | 22271.70 | 216.97 |
| GNQVSNFLANAIK | 0 | 1374.73 | 17598.74 | 44231.65 | 238.51 |
| KFFSSENLAGMFK | 1 | 1504.74 | 0.00 | 1444.30 | 62.14 |
| KNFPPFANSASSDFQYAFK | 1 | 2165.02 | 1251.05 | 3603.80 | 84.49 |
| QGSMPNQMEMVR | 0 | 1406.61 | 0.00 | 607.15 | 75.51 |
| QGSMPNQMEMVRR | 1 | 1562.71 | 10076.22 | 19121.56 | 134.99 |
| TLIWQMASGLYQDK | 0 | 1652.82 | 9714.81 | 15483.13 | 166.62 |
| TLIWQMASGLYQDKAK | 1 | 1851.96 | 0.00 | 1461.44 | 63.71 |
|  |  |  | 74109.4 | 228935.9 |  |
